# Low protein intake, muscle strength and physical performance in the very old: The Newcastle 85+ Study

**DOI:** 10.1016/j.clnu.2017.11.005

**Published:** 2018-12

**Authors:** Antoneta Granic, Nuno Mendonça, Avan Aihie Sayer, Tom R. Hill, Karen Davies, Ashley Adamson, Mario Siervo, John C. Mathers, Carol Jagger

**Affiliations:** a AGE Research Group, Institute of Neuroscience, Newcastle University, Newcastle upon Tyne, United Kingdom; b NIHR Newcastle Biomedical Research Centre, Newcastle upon Tyne Hospitals NHS Foundation Trust and Newcastle University, Newcastle upon Tyne, United Kingdom; c Newcastle University Institute for Ageing, Newcastle upon Tyne, United Kingdom; d Human Nutrition Research Centre, Newcastle University, Newcastle upon Tyne, United Kingdom; e School of Agriculture, Food and Rural Development, Newcastle University, Newcastle upon Tyne, United Kingdom; f MRC Lifecourse Epidemiology Unit, University of Southampton, Southampton, United Kingdom; g Academic Geriatric Medicine, Faculty of Medicine, University of Southampton, Southampton, United Kingdom; h Institute of Health & Society, Newcastle University, Newcastle upon Tyne, United Kingdom; i Institute of Cellular Medicine, Newcastle University, Newcastle upon Tyne, United Kingdom

**Keywords:** Aged 80 and over, Low protein intake, Newcastle 85+ Study, Grip strength, Timed Up-and-Go test, Physical activity, BMR_est_, estimated basal metabolic rate, BW, body weigh, CV, coefficient of variation, EI, energy intake, FM, fat mass, FFM, fat-free-mass, GPrr, general practice record review, GDS, Geriatric Depression Scale, GS, grip strength, (I)ADL, (instrumental) activities of daily living, IAAO, indicator amino acid oxidation, MPS, muscle protein synthesis, PA, physical activity, RDA, Recommended Dietary Allowance, SMMSE, Standardised Mini Mental State Examination, TUG, Timed Up-and-Go test, 24-hr MPR, 24 hour multiple-pass recall

## Abstract

**Background:**

Low protein intake has been linked to reduced muscle strength and physical performance in older adults but little is known about how it may affect muscle health and subsequent functional decline in the very old (aged 85+), who are at enhanced risk of malnutrition and loss of muscle mass and strength.

**Aims:**

To investigate the associations between low protein intake, defined as the intake of <1 g protein/kg adjusted body weight/day (<1 g/kg aBW/d) and decline in muscle strength and physical performance in the very old.

**Methods:**

The analytic sample consisted of 722 community-dwelling participants (60% women) from the Newcastle 85+ Study who had protein intake at baseline. Participants were followed-up for change in grip strength (GS) and Timed Up-and-Go (TUG) test over 5 years (baseline, 18, 36, and 60 months). We used mixed models to determine the effects of low protein intake on muscle strength and physical performance in all participants, and also stratified by sex.

**Results:**

At baseline, 390 (54%) participants (261 women, p < 0.001) reported low protein intake, and these differed from participants with good intake (≥1 g/kg aBW/d) on several measures of health and function. In the model adjusted for protein intake, consuming <1 g/kg aBW/d of protein was associated with a 1.62 kg lower GS (p = 0.008) in all participants, and especially in women (β (SE) = −0.83 (0.41), p = 0.05) after adjusting for key baseline covariates (anthropometry, multimorbidity, arthritis in hands, cognitive status and physical activity). The rate of decline in GS over 5 years was not associated with protein intake. Women, but not men, with low protein intake had worse baseline TUG (β (SE) = 0.04 (0.02), p = 0.03) compared with those with good protein intake in the fully adjusted model, but the rate of decline in TUG was not affected by daily protein status.

**Conclusions:**

Intake of <1 g protein/kg aBW/d may negatively affect muscle strength and physical performance in late life, especially in older women, independently of important covariates. More research is needed in the very old to define the optimal protein intake for maintenance of muscle health and function.

## 1 Introduction

Older adults experience a gradual decline in muscle mass, strength and function with ageing (sarcopenia) [1], which puts them at increased risk of falls, frailty, disability and death [2], [3]. Loss of muscle mass and strength can be further accelerated by acute and chronic stressors such as diseases, physical inactivity, bed rest, and poor diet [4], [5], [6]. Adequate intake of dietary protein has been recognised as a key modifiable factor in muscle ageing and physical decline [7], [8], [9], [10]. The muscle protein synthesis (MPS) response to protein (amino acids) ingestion is blunted in older adults [5], [6], [8], especially at lower doses [11] leading to a negative balance between skeletal MPS and muscle protein breakdown [8]. Based on epidemiological evidence, a number of recent opinion articles, reviews and expert group position papers have argued for a higher protein intake than the current Recommended Dietary Allowance (RDA) of 0.8 g/kg body weight/day (g/kg BW/d) [12] to support muscle health and function in older adults. An intake of 1.0–1.5 g/kg BW/d has been proposed as optimal for the maintenance of muscle mass, and possibly strength and function [5], [6], [9], [13], [14], [15], [16], [17], [18], although debate continues about protein requirements across age, sex, health status, body composition, and levels of physical activity (PA). For example, when establishing the prevalence of inadequate protein intake (<0.8 g/kg BW/d) in older adults living in community, Berner *et al* (2013) have emphasised the sensitivity of the protein adequacy estimates to the definition used for BW choices (actual versus ideal), and suggested that calculations should be based on BW adjusted, if necessary, for a healthy BMI [19].

The current RDA for protein (Institute of Medicine, 2005) is based on a meta-analysis of a limited number of nitrogen balance studies with short duration that included mainly healthy young adults [20]. For individuals aged 65–85 years, outcomes from metabolic studies using amino acid oxidation as an index of adequacy (the indicator amino acid oxidation (IAAO) technique) have yielded estimates of protein RDA of 1.2 g/kg BW/d for men and 1.3 g/kg BW/d for women [21]. The estimates increased to 1.6 g/kg/d in older men when based on fat-free mass (FFM). Use of the same technique in octogenarian women (aged 80–87) has suggested that 1.15 g protein/kg BW/d, or ∼30% above the current RDA, is the minimum amount required to avoid loss of lean muscle mass [22]. To ensure effective simulation of MPS and to combat muscle wasting in older adults, it has been suggested that these higher protein intakes should be achieved through intake of ∼25–30 g of protein per eating occasion across 3 main meals/d [5], [6], [8], [11].

The experts also debated that optimal protein intake in older adults should be based on optimising long-term changes in clinical and functional outcomes such as decline in lean body mass, muscle strength (grip strength, GS), and physical performance (gait speed) [5], [7], [8], [9], [10], [15], [17]. However, the evidence from prospective studies of community-dwelling older adults (aged ≥65) of the impact of dietary protein on these outcomes remains inconclusive [23], [24], [25], [26], [27], [28]. At present, there is a major evidence gap with no studies that have attempted to estimate protein needs of the very old (aged ≥85), who are at the highest risk of sarcopenia [2], [3], [29], disability [30], multimorbidity [31], inactivity, and poor nutrition [32]. Therefore, the main aim of this study was to explore the associations between low protein intake and muscle strength (GS) and physical performance (Timed Up-and-Go test, TUG) in the very old over 5 years. Secondly, we investigated whether PA and protein intake distribution across the day influenced these relationships.

## 2 Materials and methods

### 2.1 Study design and participants

The Newcastle 85+ Study is a prospective cohort study that included over 1000 participants born in 1921 who lived in Newcastle and North Tyneside, Northeast UK. The study details have been described previously [33], [34]. The study measured a range of bio-psycho-social factors that affect health and functioning of adults aged 85+ over 5 years. The analytic sample for this study comprised of 722 community-dwelling participants (289 [40%] men, and 433 [60%] women) with measure of protein intake (g/kg BW)/day), multidimensional health assessment and general practice record review (GPrr). Trained research nurses assessed participants for health and functioning (including GS and TUG) at baseline (2006/07) and follow-up at 18 (1.5 years), 36 (3 years), and 60 months (5 years) at their usual place of residence. At baseline, 713 participants (98.8%) with measured protein intake had a complete GS measurement and 714 (98.9%) had TUG assessment.

#### 2.1.1 Ethics statement

The Newcastle & North Tyneside Local Research Ethics Committee 1 approved the study. The study was conducted in accordance with The Code of Ethics of the World Medical Association (Declaration of Helsinki). Each participant signed an informed consent, and where participants lacked capacity a signed consultee approval was obtained.

### 2.2 Study variables

#### 2.2.1 Muscle strength

GS [35] was assessed using a hand-held dynamometer (Takei A5401 digital 0–100 kg × 0.1 kd LCD) in standing position as described [36]. Briefly, participants were instructed to let their arm hang normally beside the body and to flex an elbow at ∼180° angle and to squeeze the dynamometer with the maximum force. GS was recorded twice for each hand (in kg) alternating between the hands. We calculated the mean of four GS measurements (M, standard deviation, SD) for each participant, and used it in the analysis.

#### 2.2.2 Physical performance

Physical performance was assessed by the TUG test as described previously [37]. Participants were seated on a chair with armrests and seat height 46 cm off the floor. They were instructed to get up and walk as quickly as possible in a straight line up to and around a floor marker placed 3 m away, walk back, and to resume the seating position. The time needed to complete the test (from rising from the chair to sitting back down on it) was recorded in seconds (s), and the use of walking aids (e.g. cane, wheeled walker, and walking frame) was recorded.

Both GS and TUG were assessed at baseline, 18-, 36-, and 60-months follow-up.

#### 2.2.3 Protein intake and protein intake distribution

Protein intake was estimated with a validated 24-hr multiple-pass dietary recall (24-h MPR) at baseline as described previously [38], [39]. Briefly, a detailed intake of foods consumed on the previous day was recorded for each participant on two non-consecutive days of the week (excluding Fridays and Saturdays and at least one week apart) by research nurses. A unique food code was assigned to each food and mean 2-day intakes were entered in a Microsoft Access-based dietary data system. The codes were further grouped into 118 food groups based on McCance and Widdowson's composition-of-foods 6th edition [39], [40].

Low protein intake was defined as intake of <1 g protein/adjusted (ideal) BW/day (<1 g/kg aBW/d) as described in Berner *et al.* [19]. Briefly, the actual body weight was adjusted to a desirable body weight which was associated with a reduced risk of mortality for older adults aged ≥71 years if an individual was outside of a healthy BMI range of 22.0–27.0. aBW was then used to establish protein intake status (low versus good). In the analytic sample (n = 722), body weight was adjusted for 405 (56.1%) participants (256 women, p = 0.05).

To determine protein intake distribution throughout the day, meal times were grouped into 3-h slots (5:30–8:29, 8:30–11:29; 11:30–14:29; 14:30–17:29, 17:30–20:29 and 20:30–23:29) and one 6-h (overnight) slot (23:30–5:29) [41]. Protein intake distribution was calculated using the coefficient of variation (CV) (SD of protein intake between the time categories/total protein intake) excluding occasions where energy intake was 0, and categorised as (a) below CV mean of 0.187 (SD = 0.058) or 18.7% indicating more even protein intake distribution, and (b) above 18.7% representing skewed (pulse) protein intake [41].

#### 2.2.4 Covariates

In the multivariable analyses the following covariates were included: (a) sociodemographic (sex); (b) anthropometry (FFM [continuous], height [continuous]); (c) health-related factors (multimorbidity [continuous] reported from GPrr; self-rated health compared with others of the same age [excellent or very good/good/fair or poor]; cognitive impairment [<26 points on Standardised Mini Mental State Examination, SMMSE; yes/no]; arthritis in hands [any hand or both; yes/no]); (d) lifestyle (PA [low (score 0–1)/moderate (score 2–6)/high (score 7–18)]); (e) diet-related factors (protein intake distribution [even/skewed], as described above), misreporting of food intake [yes/no]); (f) use of walking aids (yes/no), and (g) attrition (completed the study/dropped out [by withdrawal or death]).

Self-reported PA was measured with a purpose-designed PA questionnaire (available at http://research.ncl.ac.uk/85plus/). Participants were classified into three categories based on the PA scores (range 0–18) derived from the frequency and intensity of PA performed in their daily life. Briefly, participants were asked about engaging in activities that were either highly energetic (e.g. swimming, cycling, heavy gardening), moderately energetic (e.g. heavy housework, moderate gardening, walking at moderate pace), and mildly energetic (e.g. light gardening, light housework), and performed at the frequency of ≥3 times/week, 1–2 times/week, 1–3 times/month, and hardly ever/never. This categorisation correlated highly with accelerometry data from this cohort [42]. Multimorbidity included cardiovascular disease (hypertension, cardiac disease), respiratory diseases, cerebrovascular diseases, diabetes, arthritis, and cancer [34]. FFM was determined by bioimpedance (Tanita Corp., Tokyo, Japan). All covariates were measured at baseline (except the use of walking aids), and FFM and height were centred to sex-specific mean. Misreporting of diet was determined based on cut-offs defined by Goldberg et al. [43] and the Fredrix equation for calculation of estimated basal metabolic rate (BMR_est_) [44] for each participant as described previously [32]. An energy intake (EI):BMR_est_ ratio below 1.05 was used to define under-reporters, and a ratio over 2.0 defined over-reporters of food intake [32].

Additional variables used as confounders (sensitivity analysis) and to describe participants by protein intake were: education (0–9/10–11/≥12 years), marital status (married/not married), social class (higher managerial and administrative/intermediate/manual and routine occupations) coded to the National Statistics Socio-economic Classification System, smoking (never/current smoker/former smoker), current alcohol intake (yes/no), presence of depressive symptoms (none [score 0–5]/mild or moderate [score 6–7]/severe [score 8–15] assessed by the Geriatric Depression Scale (GDS-15), number of difficulties with basic and instrumental activities of daily living, (I)ADLs (independent/1–6/7–12/13–17), fat mass, FM (kg), diet change in past year (yes/no) and total energy intake (kJ). All confounders were baseline variables.

### 2.3 Statistical analysis

#### 2.3.1 Descriptive statistics

Participants were compared on key sociodemographic, anthropometry, lifestyle, and health- and diet-related variables by protein intake groups (low [<1 g/kg aBW/d] versus good [≥1 g/kg aBW/d] protein intake) using the Student *t*-test (normally distributed data), Mann–Whitney *U* test (ordered and non-normally distributed data), and the Chi-square test (categorical variables) (Table 1).

**Table 1:** Characteristics of study participants by protein intake groups at baseline.

Characteristic	All participants	Low protein intake	Good protein intake	p
	n = 722	n = 390	n = 332	
*Socio-demographic factors*				
Women % (n)	60.0 (433)	66.9 (261)	51.8 (172)	<0.001
Men % (n)	40.0 (289)	33.1 (129)	48.2 (160)	
Marital status % (n)				0.12
Not married	68.6 (495)	71.8 (280)	64.7 (215)	
Married	31.4 (227)	28.2 (110)	35.2 (117)	
Years of education % (n)				0.009^a^
0–9	63.8 (458)	68.0 (264)	58.8 (194)	
10–11	23.7 (170)	21.4 (83)	26.4 (87)	
≥12	12.5 (90)	10.6 (41)	14.8 (49)	
Occupational class % (n)				0.66
Routine/manual professions	50.3 (349)	51.9 (194)	48.4 (155)	
Intermediate professions	14.6 (101)	13.9 (52)	15.3 (49)	
Higher managerial/administrative	35.2 (244)	34.1 (128)	36.3 (116)	
*Diet-related factors*				
Diet change in past year % (n)				0.37
Yes	6.7 (48)	7.4 (29)	5.8 (19)	
No	93.3 (668)	53.7 (359)	46.3 (309)	
Total energy, kJ (M, SD)	7030.0 (2158.8)	6142.4 (1736.5)	8123.05 (2038.4)	<0.001^a^
Protein intake distribution^b^ % (n)				0.02
Even (below CV mean: <18.7%)	52.8 (381)	58.3 (222)	41.7 (159)	
Skewed (above CV mean: >18.7%)	47.2 (341)	49.3 (169)	50.7 (173)*	
Misreporting food intake^c^ % (n)				<0.001
No	82.8 (598)	49.7 (297)	50.3 (301)	
Yes	17.2 (124)	75.0 (93)	25.0 (31)*	
*Lifestyle factors*				
Smoking % (n)				0.2
Never	33.7 (243)	31.6 (123)	36.1 (120)	
Current smoker	6.0 (43)	5.1 (20)	6.9 (23)	
Former smoker	60.3 (435	63.2 (246)	56.9 (189)	
Current alcohol intake % (n)				0.13
Yes	62.7 (453)	60.3 (235)	65.7 (218)	
No	37.3 (269)	42.4 (155)	57.6 (114)	
Physical activity (PA)^d^ % (n)				0.06^a^
Low (score 0–1)	17.5 (126)	18.5 (72)	16.3 (54)	
Moderate (score 2–6)	45.2 (326)	47.7 (186)	42.3 (140)	
High (score 7–18)	37.3 (269)	33.8 (132)	41.4 (137)	
*Health-related factors*				
Self-rated health				0.001
Excellent/very good	41.3 (296)	37.3 (144)	45.9 (152)	
Good	37.2 (267)	36.0 (139)	38.7 (128)	
Fair/poor	21.5 (154)	26.7 (103)	15.4 (51)	
Multimorbidity (M, SD)	2.24 (1.23)	2.28 (1.21)	2.19 (1.25)	0.32
Number of difficulties with (I) ADLs % (n)				
Independent	21.9 (158)	17.4 (68)	27.1 (90)	0.02^a^
1–6	56.0 (404)	59.7 (233)	51.5 (171)	
7–12	18.3 (132)	17.9 (70)	18.7 (62)	
13–17	3.9 (28)	4.9 (19)	2.7 (9)	
Depressive symptoms^e^% (n)				
0–5/none	79.0 (568)	78.1 (300)	83.2 (268)	0.08^a^
6–7/mild	12.1 (87)	13.5 (52)	10.9 (35)	
≥8/severe	7.1 (51)	8.2 (32)	5.9 (19)	
Cognitive status % (n)				0.23
Normal (26–30 SMMSE score)	77.1 (556)	75.4 (294)	79.2 (262)	
Normal (≤25 SMMSE score)	22.9 (165)	24.6 (96)	20.8 (69)	
Arthritis in hands % (n)				0.86
Yes	6.5 (47)	6.7 (26)	6.4 (21)	
No	93.5 (672)	93.3 (363)	93.6 (309)	
*Anthropometry*				
Height (M, SD)	161.70 (7.65)	160.83 (7.63)	162.71 (7.57)	0.001^f^
FFM (M, SD)	45.17 (9.03)	45.00 (9.32)	45.42 (8.68)	0.51
FM (M, SD)	18.86 (7.71)	20.22 (8.07)	17.4 (7.02)	<0.001^a^
*Attrition* % (n)				0.06
Completed the study	45.3 (327)	42.1 (164)	49.1 (163)	
Dropped out (withdrawal and death)	54.7 (395)	57.2 (226)	42.8 (169)	

(I)ADLs, basic and instrumental activities of daily living; M, mean; SD, standard deviation; SMMSE, Standardized Mini-Mental State Examination; FM, fat mass; FFM, fat-free mass.

a Mann-Whitney *U* test for ordered and non-normally distributed continuous variables.

b Protein distribution was determined by the coefficient of variation (CV) calculation (ratio of SD of protein intake between time categories and mean (total) protein intake). Values below CV mean of 0.187 (18.7%) represented more even, and above 18.7% represented more skewed (pulse) protein intake throughout the day.

c Mis-reporters of food intake were determined as described previously [32].

d Based on a purpose-designed PA questionnaire assessing the type and amount of PA performed in daily life.

e Fifteen point Geriatric Depression Scale (GDS).

f Student *t*-test for normally distributed data. The χ^2^ test was used for all other categorical variables. *Adjusted residuals were used to for the *post hoc* χ^2^ test analyses at α = 0.05.

#### 2.3.2 Muscle strength (GS) and physical performance (TUG) by protein intake

The same analyses were used to describe the low and good protein intake groups in respect of untransformed GS (kg) and TUG times (s) at baseline and at each follow-up (Table 2).

**Table 2:** Grip strength and Timed Up-and-Go untransformed scores by protein intake groups at baseline and follow-up.

Physical performance/scores	All participants	Low protein intake	Good protein intake	p^a^
	n = 722	n = 390	n = 332	
*Grip strength*				
Baseline (n)	713	385	328	
kg (M, SD)	18.20 (7.08)	17.15 (7.64)	19.43 (7.33)	<0.001
Follow-up at 1.5 years (n)	567	294	273	
kg (M, SD)	17.37 (7.64)	16.52 (7.67)	18.28 (7.52)	<0.001
Follow-up at 3 years (n)	430	227	203	
kg (M, SD)	16.58 (7.28)	16.14 (7.49)	17.08 (7.04)	0.18
Follow-up at 5 years (n)	286	139	147	
kg (M, SD)	14.91 (7.04)	14.32 (7.19)	15.48 (6.87)	0.17
*Timed Up-and-Go (TUG) and use of walking aids*				
Baseline (n)	714	383	331	
s (M, SD)	18.70 (13.66)	19.08 (14.78)	16.92 (12.15)	0.001
Use of walking aids, % (n) yes	17.1 (122)	19.3 (74)	14.5 (48)	0.09
Follow-up at 1.5 years (n)	530	274	254	
s (M, SD)	21.03 (15.06)	22.64 (16.15)	19.32 (13.62)	0.001
Use of walking aids, % (n) yes	16.4 (87)	19.8 (54)	12.8 (33)	0.03
Follow-up at 3 years (n)	386	199	187	
s (M, SD)	20.37 (13.82)	21.80 (13.93)	18.85 (13.57)	0.001
Use of walking aids, % (n) yes	16.9 (65)	21.2 (42)	12.3 (23)	0.02
Follow-up at 5 years (n)	267	137	133	
s (M, SD)	20.75 (12.05)	21.29 (11.17)	20.21 (12.89)	0.16
Use of walking aids, % (n) yes	26.1 (70)	26.9 (36)	25.4 (34)	0.78

a Student *t*-test for normally distributed continuous variables, Mann–Whitney *U* test for non-normally distributed continuous data (untransformed), and χ^2^ test for categorical variables. Only significant p values at α ≤ 0.05 are reported.

#### 2.3.3 Change in muscle strength (GS) and physical performance (TUG) by protein intake

GS data (kg) were normally distributed. TUG times (s) were positively skewed at each measurement, and were log_10_ transformed, and used as a continuous variable. Lower log_10_-s indicated quicker (better) performance in TUG test.

We used mixed models [45] to determine: (a) the association between protein intake (low versus good) and GS and TUG times at baseline and over time adjusting for key confounders reported in the literature and established previously [36]; and (b) stratified by sex and (c) protein intake groups (both GS and TUG) in supplementary analysis.

For GS we fitted the following linear growth curve models: (a) with ‘time’ in study (continuous) at Level 1 (within-person level) to examine the linear trend of time, and protein intake at Level 2 (between-person level) to examine whether initial status (intercept) varied by protein intake (Model 1); (b) including an interaction between protein intake and time, to examine the rate of change (varying slopes) by protein intake groups (Model 2); and (c) adjusting for confounders (Model 3 [sex, anthropometry, health-related variables, PA, attrition] and interaction terms (sex × time, PA × time) (Table 3). Random effects terms in the models included both GS intercept and GS slope over time.

**Table 3:** β coefficients^a^ of growth curve models for grip strength (GS) over 5-year follow-up by protein intake groups.

Outcome	Effects/variable	Model 1		Model 2		Model 3	
		β (SE)	p	β (SE)	p	β (SE)	p
GS (kg)	Intercept	19.29 (0.45)	<0.001	19.50 (0.47)	<0.001	7.0 (1.25)	<0.001
*All participants*	Protein intake group						
	Low protein	−1.62 (0.60)	0.008	−2.03 (0.65)	0.002	−0.27 (0.41)	0.51
	Decline						
	Time	−0.76 (0.05)	<0.001	−0.86 (0.08)	<0.001	−0.94 (0.2)	<0.001
	Slopes (rate of decline)						
	Protein intake group × Time						
	Low protein × Time			0.19 (0.11)	0.08	0.07 (0.11)	0.49
GS (kg)	Intercept	25.30 (0.60)	<0.001	25.35 (0.61)	<0.001	13.73 (3.01)	<0.001
*Men*	Protein intake group						
	Low protein	0.65 (0.90)	0.47	0.54 (0.92)	0.56	0.79 (0.84)	0.35
	Decline						
	Time	−1.13 (0.10)	<0.001	−1.18 (0.13)	<0.001	−2.02 (0.36)	<0.001
	Slopes						
	Protein intake group × Time						
	Low protein × Time		0.61	−0.13 (0.21)	0.54	0.1 (0.20)	0.6
GS (kg)	Intercept	14.54 (0.33)	<0.001	14.6 (0.34)	<0.001	8.8 (1.19)	<0.001
*Women*	Low protein intake group						
	Low protein	−0.93 (0.41)	0.02	−1.05 (0.45)	0.02	−0.83 (0.41)	0.046
	Decline						
	Time	−0.53 (0.06)	<0.001	−0.57 (0.08)	<0.001	−0.63 (0.21)	0.003
	Slopes						
	Protein intake group × Time						
	Low protein × Time			0.07 (0.11)	0.53	0.06 (0.12)	0.59

Model 1 includes a linear trend of time and protein intake group at baseline.

Model 2 includes protein intake and time interaction term.

Model 3 is additionally adjusted for sex (in all participants), anthropometry (height and FFM), health-related factors (number of chronic diseases, self-rated health, cognitive impairment, arthritis in hands), PA, attrition variable, and interaction terms (sex × time, PA × time).

a Parameter estimates β coefficients (SE) of fixed effects with GS longitudinal data. Random effects included both intercept and slopes of GS over 5 years. Time was used as continuous variable. Good protein intake (≥1 g/kg adjusted BW/day) served as a reference group.

In the models with TUG we included both linear and quadratic effects of time (i.e. to account for nonlinear change over 5 years) and protein intake × time interaction term, and adjusted for similar confounders and included diet-related factors (protein distribution and misreporting of food intake), and use of walking aids (Table 4). All confounders were time-invariant (baseline) variables except for walking aids use (Table 1). Random effects in the models included both intercept and slope (linear) of TUG over time.

**Table 4:** β coefficients^a^ of growth curve models for Timed up-and-go (TUG) test over 5-year follow-up by protein intake groups.

Outcome	Effects/variable	Model 1		Model 2		Model 3	
		β (SE)	p	β (SE)	p	β (SE)	p
TUG (log_10_-s)	Intercept	1.12 (0.01)	<0.001	1.11 (0.01)	<0.001	1.41 (0.04)	<0.001
*All participants*	Protein intake group						
	Low protein	0.05 (0.02)	0.001	0.05 (0.02)	0.002	0.03 (0.01)	0.04
	Decline						
	Time	0.02 (0.002)	<0.001	0.05 (0.006)	<0.001	0.05 (0.006)	<0.001
	Time^2^			−0.008 (0.001)	<0.001	−0.008 (0.001)	<0.001
	Slopes (rate of decline)						
	Protein intake group × Time						
	Low protein × Time			0.007 (0.009)	0.42	0.007 (0.009)	0.40
	Low protein × Time^2^			−0.002 (0.002)	0.21	−0.002 (0.002)	0.21
TUG (log_10_-s)	Intercept	1.10 (0.02)	<0.001	1.09 (0.02)	<0.001	1.36 (0.06)	<0.001
*Men*	Protein intake group						
	Low protein	0.02 (0.02)	0.3	0.02 (0.02)	0.44	0.01 (0.02)	0.51
	Decline						
	Time	0.02 (0.003)	<0.001	0.05 (0.009)	<0.001	0.06 (0.009)	<0.001
	Time^2^			−0.007 (0.002)	<0.001	−0.01 (0.002)	<0.001
	Slopes						
	Protein intake group × Time						
	Low protein × Time			0.01 (0.01)	0.45	−0.004 (0.01)	0.75
	Low protein × Time^2^			−0.002 (0.003)	0.41	0.001 (0.003)	0.81
TUG (log_10_-s)	Intercept	1.14 (0.02)	<0.001	1.13 (0.02)	<0.001	1.41 (0.04)	<0.001
*Women*	Protein intake group						
	Low protein	0.06 (0.02)	0.005	0.06 (0.02)	0.005	0.04 (0.02)	0.03
	Decline						
	Time	0.02 (0.002)	<0.001	0.06 (0.008)	<0.001	0.05 (0.008)	<0.001
	Time^2^			−0.008 (0.002)	<0.001	−0.007 (0.002)	<0.001
	Slopes						
	Protein intake group × Time						
	Low protein × Time			0.006 (0.01)	0.61	0.01 (0.01)	0.24
	Low protein × Time^2^			−0.003 (0.002)	0.3	−0.004 (0.02)	0.1

Model 1 includes a linear trend of time and protein intake group at baseline.

Model 2 includes, in addition, a quadratic trend of time, and protein intake and time interaction terms (both linear and quadratic).

Model 3 is additionally adjusted for sex, anthropometry (height and FFM), health-related factors (number of chronic diseases, self-rated health, cognitive impairment), PA, attrition variable, protein distribution, food intake misreporting, and use of walking aids at baseline and follow-up.

a Parameter estimates β coefficients (SE) of fixed effects with TUG longitudinal data (log_10_-transformed). Random effects included both intercept and slopes of TUG times (log_10_-transformed) over 5 years. Time was used as continuous variable. Good protein intake (≥1 g/kg adjusted BW/day) served as a reference group.

Negative β estimates for GS, and positive (increasing) β estimates for TUG represent poor performance. SPSS MIXED procedure (SPSS, 2002), restricted maximum likelihood (RML), and unstructured covariance matrix was used to create parameter estimates (β) for both outcomes.

### 2.4 Sensitivity and supplementary analyses

In preliminary analysis, we first fitted linear growth curve models with low protein intake defined as <0.8 g/kg aBW/d to investigate the association with GS and TUG at baseline and over time (details not shown). GS models with low protein intake of <1 g/kg aBW/d were additionally adjusted for diet-related variables (protein distribution, misreporting of food intake, diet change in past year, and total energy intake), and TUG models for diet change and total energy intake in all participants, men and women. Separate (supplementary) analyses for GS and TUG change over time were conducted after stratification by protein intake (low [<1 g/kg aBW/d] versus good [≥1 g/kg aBW/d]) and adjusted for the same set of covariates in reported (Supplementary Tables 1 and 2) and sensitivity analysis (details not shown).

We used IBM SPSS (V.21; IBM Corporation, Armonk, NY, USA) to conduct the analyses, and 2-sided statistics at α = 0.05 statistical significance.

## 3 Results

Baseline characteristics of 722 participants with protein intake status (low versus good) are presented in Table 1. The groups differed on key sociodemographic and health measures. Specifically, participants with low protein intake were more likely to be women (p < 0.001), less educated (p = 0.009), had more difficulties with (I)ADLs (p = 0.02) and were more likely to report fair/poor health (p = 0.001). Participants with good protein intake had lower FM (p < 0.001), consumed more total energy from diet (p < 0.001), were more likely to have skewed protein distribution (p = 0.02), but less likely to misreport food intake (p < 0.001) compared with those with low protein intake.

### 3.1 Grip strength by protein intake over 5 years

Untransformed values for GS (kg) and TUG (s) by protein intake at baseline and follow-up are presented in Table 2. Those with good protein intake (≥1 g/kg aBW/d) had stronger GS at baseline and at 1.5-year follow-up, and lower (quicker) TUG times (s) at baseline, 1.5- and 3-year follow-up.

**Fig. 1:**
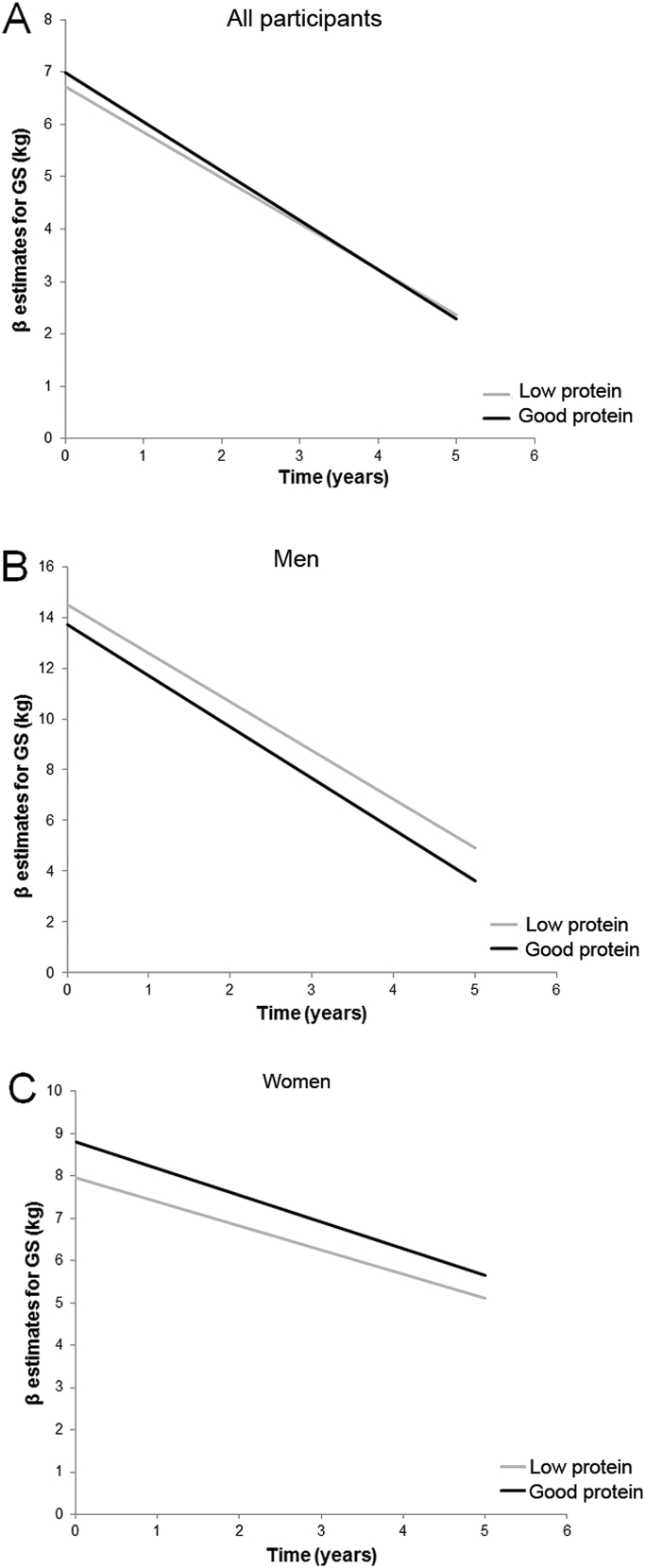
**A similar rate of decline in GS by protein intake**. We found no association between low protein intake (<1 g/kg aBW/d; grey line) and grip strength (GS) at baseline or GS decline compared with good protein intake (≥1 g/kg aBW/d; black line) in all participants (panel A) and in men (panel B). In women (panel C), low protein intake (grey line) was associated with lower GS at baseline, but not with the rate of GS decline over 5 years. The growth curves represent β estimates of the fully adjusted model (Model 3). Greater β estimates indicate higher muscle strength (GS).

The results of mixed models examining the association between protein intake and GS change over 5 years are shown in Table 3. In the analysis with all participants (n = 722), GS declined significantly over the study period (p < 0.001 in all models), and particularly in men (p < 0.001). Specifically, GS declined linearly by −0.76 kg per year in all, and by −1.13 and −0.53 kg per year in men and women, respectively (Model 1). When tested as a main effect (Model 2), low protein intake was associated with lower GS (β [SE] = −2.03 [0.65], p = 0.002) at baseline, but not with the rate GS decline over 5 years (p = 0.08). After adjustment for key covariates (sex, FFM, height, multimorbidity, self-rated health, cognitive status, arthritis in hands, PA, attrition and interaction terms) (Model 3), significant associations with baseline GS and low protein intake remained only in women (−0.83 [0.41], p = 0.046) (Fig. 1, panel C).

The rates of GS decline (slopes) did not vary by protein intake over 5 years (Fig. 1), but they varied by the levels of PA in all participants (high: 0.42 (0.21), p = 0.04), and men (high: 1.0 (0.38), p = 0.01), but not in women (high: −0.02 (0.23), p = 0.95) compared with those with low PA (Model 3, details not shown).

### 3.2 Timed Up-and-Go test by protein intake over 5 years

**Fig. 2:**
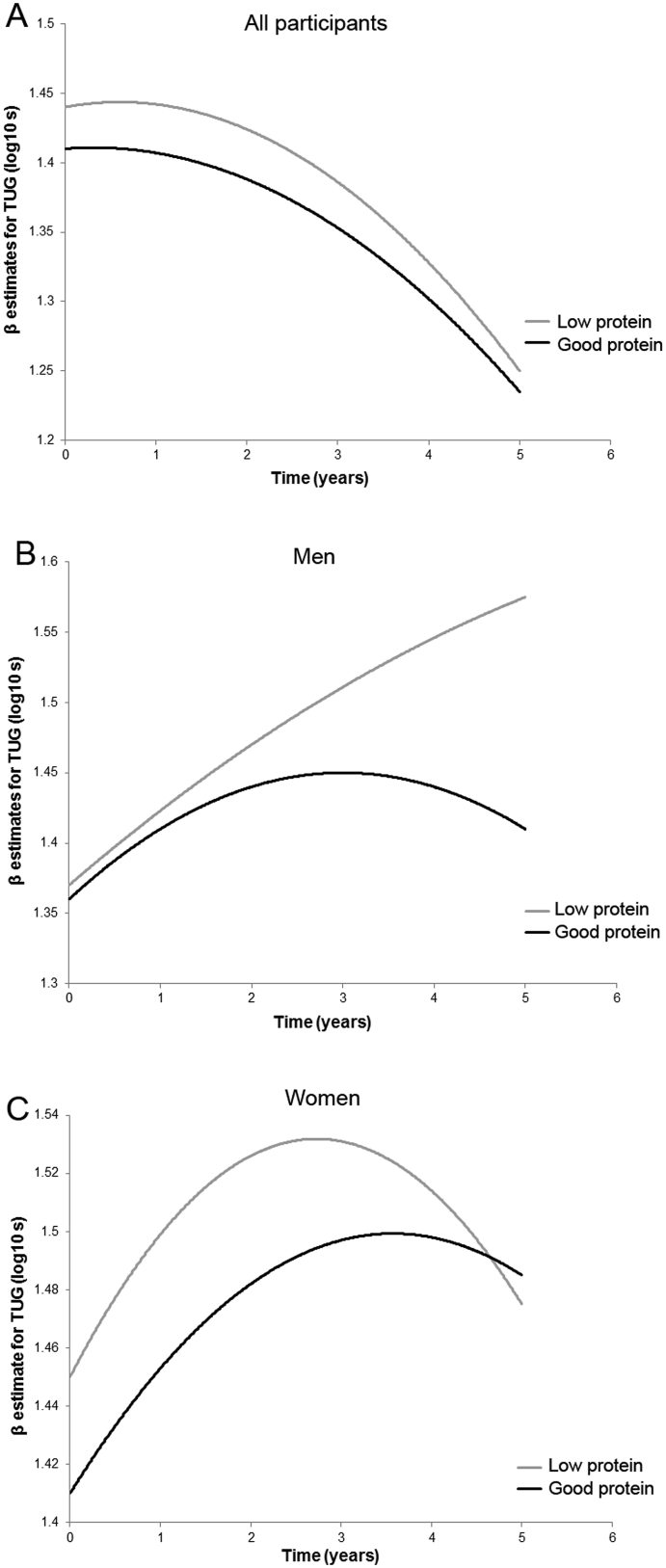
**A similar rate of decline in TUG by protein intake**. Low protein intake (<1 g/kg aBW/d; grey line) was associated with worse Timed Up-and-Go (TUG) test scores at baseline in all participants (panel A) and in women (panel C), but not in men (panel B) compared with those consuming ≥1 g/kg aBW/d (black lines) of protein. The rate of decline in TUG did not vary by protein intake (panels A, B, and C) over 5 years. The growth curves represent β estimates of the fully adjusted model (Model 3). Greater log_10_-s indicated worse (slower) TUG performance.

We observed a significant linear decline in TUG (log_10_-transformed) means (log_10_-s) over 5 years follow-up, representing poorer (slower) performance in all participants, men and women (p < 0.001 in all) (Model 1 to 3, Table 4). The overall average TUG speed declined by 0.03 log_10_-s/year after adjustment for covariates, and by 0.06 log_10_-s in men, and 0.05 log_10_-s in women (Model 3, Table 4). A significant (although small) quadratic effect of time was observed in all participants (β [SE] = −0.008 [0.001]), men (−0.01 [0.002]), and women (−0.007 [0.002]) (p < 0.001 for all) (Model 3), suggesting a deceleration in the rate of change in TUG over 5 years (Fig. 2).

In the fully adjusted model (Model 3) which included diet-related factors (protein intake distribution and misreporting of food intake), low protein intake was associated with slower TUG times at baseline (0.04 [0.02], p = 0.03) in women, but not in men. The rate of decline in TUG (slope) over 5 years was not associated with protein intake (Table 4, Fig. 2). Both high and moderate PA was a significant predictor of TUG at baseline but not over time in all participants (high: −0.16 [0.22], p < 0.001; moderate: −0.08 [0.02], p < 0.001, Model 3), men and women (details not shown).

### 3.3 Results for sensitivity and supplementary analyses

We found no significant associations between low protein intake defined as <0.8 g/kg aBW/d and GS and TUG (initially and over time), confirming that this cut-off may be too low for optimal health outcomes in the very old (details not shown) [20], [21].

The association between low protein intake defined as <1 g/kg aBW/d and baseline GS (Model 3, Table 3) in women was attenuated to non-significant after adjustment for protein intake distribution, misreporting of food intake, and total energy intake (−0.80 [0.45], p = 0.08; details not shown), although neither covariate was a significant predictor of GS. However, the association remained significant when FM was used as a confounder instead of FFM (−0.93 [0.46], p = 0.04; details not shown). Adding all diet-related variables to the final GS model (Model 3, Table 3) did not change the conclusions (e.g. low protein intake was not associated with GS initially and over time in all participants, but high PA remained significant predictors of GS change over 5 years in all participants and in men [0.42 (0.21), p = 0.04; 1.00 (0.38), p = 0.01, respectively], but not in women [−0.03 (0.23), p = 0.92]. Similarly, adding diet change in the past year and total energy to the final TUG models (Model 3, Table 4) did not change the findings (details not shown).

We fitted multi-level models for GS and TUG stratified by protein intake group (low versus good protein intake) to investigate the factors associated with initial level and change (decline) in GS and TUG over 5 years (Supplementary Tables 1 and 2, respectively). We reported only significant associations for key covariates. In the fully adjusted model (Model 3, Supplementary Table 1) we observed a non-significant effect of time in the low protein group, indicating little change in GS over time. Significant predictors of higher baseline GS in this group were sex (p < 0.001), moderate and high PA, higher FFM (all p < 0.001), height (p = 0.001), excellent/very good health (p = 0.04), not having arthritis in hands (p < 0.001), and completing the study (p = 0.02).

For those in the good protein intake group, comparable covariates were significant predictors of higher GS at baseline (e.g. sex, height, FFM, high PA), including having no cognitive impairment (p = 0.03). Men experienced a greater rate of annual decline in GS (−0.62 [0.16], p < 0.001) compared with women, but those reporting high and moderate levels of PA had less steep slopes compared with their non-active counterparts (by 0.83 kg and 0.77 kg/year, respectively) (Supplementary Fig. 1).

Similarly, significant predictors of better baseline performance in the TUG test in the low protein intake group were sex (men), PA (moderate and high), self-rated health (excellent/very good and good), and not using walking aids (Supplementary Table 2). Whilst having no cognitive impairment (but not sex and self-rated health) predicted better baseline TUG times in the good protein intake group. Higher disease count (multimorbidity) was associated with worse TUG performance in both groups.

Sex and PA were not significant predictors of GS or TUG slopes (i.e. interaction terms sex × time, and PA × time were not significant). Protein intake distribution was not associated with either GS or TUG at baseline or with rate of decline in GS or in TUG over 5 years, and adding total energy intake to the models did not change the findings (details not shown).

## 4 Discussion

We investigated the relationships between low protein intake (<1 g protein/kg adjusted (ideal) body weight/day; <1 g/kg aBW/d) and muscle strength (GS) and physical performance (TUG) at age 85 years (baseline) and decline in these outcomes over 5 years in participants from the Newcastle 85+ Study living in the community. To the best of our knowledge, this is the first such prospective investigation of the effect of low protein intake on GS and TUG in the very old. Daily intake of protein of <1 g/kg aBW was associated with a 0.83 kg lower GS and worse TUG performance at baseline in women after adjustment for a range of confounders, but the subsequent rates of decline in GS and in TUG were not affected by protein intake. Protein distribution intake throughout the day (even versus skewed) and total energy were not associated with GS and TUG. Higher physical activity (PA) was a significant predictor of slower GS decline in all participants, in those with good protein intake (≥1 g/kg aBW/d), and in men but not in women.

Optimal protein intake is essential for the maintenance of good health and lean muscle mass across all ages. The current RDA (0.8 g/kg BW/d) [12] is the same for younger and older healthy adults and reflects the minimum amount of protein required to sustain nitrogen balance regardless of sex, body composition, metabolic changes, health status and activity level [20], [21]. Several recent expert opinions and consensus statements have highlighted evidence that higher protein intake (1–1.5 g/kg BW/d) may be needed in older adults to promote healthy ageing and to sustain function [5], [6], [9], [13], [14], [15], [16], [17], [18]. Evidence-based recommendations from the European Society for Clinical Nutrition and Metabolism [17], the European Union Geriatric Medicine Society PROT-AGE study group [15], and Protein Summit 2.0 [6] emphasised the unique features of protein needs of older adults for maintenance of muscle function which include not only (a) protein quantity, but also (b) protein distribution (pattern of intake across the day), and (c) amount of protein ingested per eating occasion in combination with (d) habitual PA and exercise. Higher protein intake (>1.0 g/kg BW/d) and ingestion of at least ∼25–30 g protein in each of three main meals in temporal proximity to PA [6] may be needed to optimally stimulate MPS in older adults. These arguments are based on evidence that older individuals have a blunted response to ingested protein at lower intakes (anabolic resistance) [5], [6], [11], adopt more skewed protein distribution throughout the day [19], and have more pro-catabolic stressors (i.e. multimorbidity and inactivity) [6], [8]. We also found no association between low protein intake characterised as <0.8 g/kg aBW/d and GS and TUG, suggesting that this cut-off may be too low for health and function in the very old (details not shown).

Observational and metabolic studies investigating the roles of these factors in establishing optimal, rather than minimal, protein intake for musculoskeletal health and function in the very old (aged ≥85) are lacking [22], [46], despite this age group being at enhanced risk of malnutrition [47], sarcopenia [29], and loss of independence [30]. Only a few prospective studies have investigated the relationships between dietary protein intake and physical performance/muscle function in older adults and none included a significant number of the very old (≥85 years). In addition, these studies have used various cut-offs to define protein adequacy which limits comparison of their observations with our findings [24], [25], [26], [27], [28], [48]. For example, in the Women's Health Initiative (a large, prospective study of postmenopausal women [∼140,000 participants] aged 50 to 79), higher biomarker-calibrated protein intake (1.18 g/kg BW/d) was associated with greater GS at baseline, and with reduced rates of decline in GS and in chair stands over a mean follow-up of 11.5 years [25]. Higher intake of total and animal protein (≥1.0 g/kg BW/d) were protective against GS loss in adults aged ≥60 in the Framingham Offspring Cohort (20% postmenopausal women) over 6 years [26], independently of lean mass. Higher protein intake (≥1.2 g/kg BW/d) was associated with better performance in several measures of muscle strength and physical performance at baseline and over 3-year follow-up in women (aged 65–72), but was dependent on fat mass [27]. Additional studies of octogenarian men and women are needed to define the optimal intake of protein for muscle strength and function and the possible impact on protein needs of differences in body composition and in health status.

Women in the present study who consumed <1.0 g/kg aBW/d of protein had 0.83 kg lower GS and 0.04 log_10_-s worse TUG at baseline compared with women with higher protein intake, irrespective of key confounders (i.e. lean mass, FM, multimorbidity, cognitive impairment, and PA). Other factors and especially high PA (but not total protein intake, protein distribution across the day or total energy intake) were associated with higher GS at baseline and slower GS decline in the entire cohort, in participants with good protein intake (≥1 g/kg aBW/d), and in men. The lack of association between GS, TUG, and protein intake in men may have several explanations. The protein cut-off that we used to define adequacy may be too low for men who had different body composition (i.e. higher FFM and lower FM) and higher energy requirements (i.e. higher PA) compared with women (data not shown). Application of the IAAO method yielded an estimate of 1.6 g/kg BW/d as optimal for older men [21]. The recent comparison of MPS between younger (∼22 years) and older men (∼71 years) set protein intake at ∼1.2 g/kg BW/d as optimal for muscle protein anabolism [11]. A single nutrient approach (protein) in examining the relationship between muscle function and diet disregards the likely synergistic/antagonistic or cumulative effect of other foods and nutrients (e.g. fat, vitamin D, n−3 fatty acids) and of the amino acid composition of ingested proteins on muscle health. Other nutrients, food groups and dietary patterns (DP) may play a greater role in determining muscle strength and physical performance in very old men. Indeed, we reported previously that, within the Newcastle 85+ Study, men in DP dominated by high intake of saturated fat spreads and oil (butter) and low intake of unsaturated fat sources experienced a faster rate of GS decline [49].

Participants in the good protein intake group who reported moderate and high PA had 2 and 4 kg higher GS at baseline and lost less GS per year (0.77 kg and 0.83 kg), respectively compared to those with low PA. However, higher PA in the low protein intake group was not protective of GS decline over time (Supplementary Fig. 1). The results indicate that higher levels of PA may be insufficient to slow age-related decline in muscle strength in older adults unless protein intake is adequate (≥1 g/kg aBW/d), although the finding may be biased (Type II error; loss of power in data), and influenced by the type of variable we used to measure PA (self-reported versus objectively measured; ordinal versus continuous variable). Equally, good protein intake may not protect against GS decline if the level of PA is too low (evident by the steepest GS slope in good protein-low PA group). Intervention studies [50] and expert groups [6], [15], [17] suggest that a synergistic action of high protein intake, exercise (e.g. resistance) and habitual PA is required to enhance MPS and to reduce muscle mass/strength loss, whilst anabolic resistance in older adults is precipitated by inactivity [8]. More research is needed to establish the benefits or clinical relevance of higher protein intake (≥1 g/kg aBW/d) in combination with PA for muscle health and function in very old adults.

This study has several limitations which may have influenced the findings: (a) use of baseline confounders and residual/unknown confounders may have affected the associations between low protein intake, GS and TUG (e.g. poor dentation [51], appetite loss, everyday emotions, social support, food accessibility and dietary knowledge [52]); (b) protein intake estimate (by 24-hr MPR at baseline) may not have captured habitual intake accurately, and misreporting of food intake (17.2% of analytic sample) may have led to misclassification of exposure; (c) attrition (mostly due to mortality) [53] and the consequential loss of power in data over the follow-up may have affected non-significant associations, and the inclusion of very robust older ‘survivors’ may have posed another source of bias, and (d) limited generalisability of the findings. The strengths of the study are: (a) 5-year follow-up of the very old; (b) objective assessments of muscle strength and function; (c) a cohort that is broadly representative of UK population of the very old; (d) validated dietary assessment [38], and (e) adjustment for several known factors associated with muscle health [36].

In conclusion, we found associations between low protein intake (<1 g/kg aBW/d) and worse baseline GS and TUG in older adults aged ≥85 independent of key risk factors related to muscle mass and function. The results need to be replicated in other populations and may provide an important foundation for dietary interventions to preserve muscle strength and physical performance in the very old, especially in women.

## Statement of authorship

AG and CJ designed the research. KD and CJ were responsible for the Newcastle 85+ Study design, management and data acquisition. AG analysed data and wrote the manuscript. AAS, NM, TRH, KD, AA, MS, JCM, and CJ revised the manuscript for intellectual content. AG had primary responsibility for final content. All authors revised and approved the submitted version of the manuscript.

## Conflict of interest statement

None declared.

## Funding

Funding for this research is provided by the European Horizon 2020 PROMISS Project ‘Prevention Of Malnutrition In Senior Subjects in the EU’, Grant agreement no. 678732 (AG, NM, CJ). The content only reflects the author's view and the Commission is not responsible for any use that may be made of the information it contains.

The core Newcastle 85+ study was supported by a joint grant from the UK Medical Research Council and the Biotechnology and Biological Sciences Research Council (grant reference G0500997), the Dunhill Medical Trust (grant reference R124/0509) and NHS North of Tyne (Newcastle Primary Care Trust). Funding sources had no role in the collection, analysis and interpretation of data, the writing, and the decision to submit this article for publication.

## Appendix A Supplementary data

mmc1
