# Supplementary material for: Low protein intake, muscle strength and physical performance in the very old: The Newcastle 85+ Study
Source: Clin Nutr. 2018 Dec;37(6Part A):2260–70. doi: 10.1016/j.clnu.2017.11.005 (PMC6295979; doi:10.1016/j.clnu.2017.11.005)
Supplement: mmc1 [file mmc1.docx]

**SUPPLEMENTARY INFORMATION**

**Supplementary figure legend**

Supplementary Figure 1. GS decline in low and good protein intake group by PA level. Participants in the good protein intake group (grey solid and grey dashed line, respectively) with high and moderate physical activity (PA) lost less grip strength (GS) per year compared with those with low levels of PA (grey dotted line). High and moderate PA was associated with higher baseline GS in the low protein group (black solid and black dashed line), but not with the rate of GS decline over 5 years. Participants in the good protein group with low levels of PA (grey dotted line) had the worst GS trajectory. The growth curves represent β estimates of the fully adjusted model (Model 3).

**

**

Supplementary Figure 1. GS decline in low and good protein intake group by PA level.

Supplementary Table 1. β coefficients* of growth curve models for grip strength (GS) over 5-year follow-up in low and good protein intake group^†^

| Outcome | Effects/variable | Model 1 |  | Model 2 |  | Model 3 |  |
| --- | --- | --- | --- | --- | --- | --- | --- |
|  |  | β (SE) | p | β (SE) | p | β (SE) | p |
| GS (kg) | Intercept | 17.39 (0.47) | <0.001 | 5.57 (1.59) | 0.001 | 3.83 (1.71) | 0.03 |
| *Low protein* | Sex |  |  |  |  |  |  |
|  | Men |  |  | 11.89 (0.6) | <0.001 | 12.0 (0.60) | <0.001 |
|  | Women (ref) |  |  | 0 |  | 0 |  |
|  | Disease count |  |  | -0.60 (0.23) | 0.008 | -0.55 (0.22) | 0.02 |
|  | Self-rated health |  |  |  |  |  |  |
|  | Excellent/very good |  |  | 1.77 (0.71) | 0.02 | 2.09 (0.71) | 0.04 |
|  | Good |  |  | 1.14 (0.70) | 0.1 | 1.17 (0.68) | 0.09 |
|  | Fair/poor (ref) |  |  | 0 |  | 0 |  |
|  | Arthritis in hands (No) |  |  | 4.16 (1.03) | <0.001 | 4.08 (1.02) | <0.001 |
|  | Yes (ref) |  |  | 0 |  | 0 |  |
|  | Height |  |  | 0.20 (0.06) | 0.001 | 0.20 (0.06) | 0.001 |
|  | Fat-free mass |  |  | 0.14 (0.04) | <0.001 | 0.16 (0.04) | <0.001 |
|  | Physical activity (PA) |  |  |  |  |  |  |
|  | High |  |  | 4.19 (0.98) | <0.001 | 4.43 (0.98) | <0.001 |
|  | Moderate |  |  | 3.91 (0.92) | <0.001 | 4.10 (0.92) | <0.001 |
|  | Low (ref) |  |  | 0 |  | 0 |  |
|  | Attrition |  |  |  |  |  |  |
|  | Completed the study |  |  | 1.37 (0.55) | 0.02 | 1.21 (0.55) | 0.02 |
|  | Dropped out (ref) |  |  | 0 |  | 0 |  |
|  | Decline |  |  |  |  |  |  |
|  | Time | -0.66 (0.07) | <0.001 | -0.19 (0.33) | 0.56 | -0.15 (0.33) | 0.65 |
|  | Slopes |  |  |  |  |  |  |
|  | Sex × Time |  |  |  |  |  |  |
|  | Men × Time |  |  | -0.53 (0.16) | 0.001 | -0.54 (0.16) | 0.001 |
|  | Women × Time (ref) |  |  | 0 |  | 0 |  |
| GS (kg) | Intercept | 19.49 (0.45) | <0.001 | 8.28 (1.92) | <0.001 | 8.48 (2.09) | <0.001 |
| *Good protein* | Sex |  |  |  |  |  |  |
|  | Men |  |  | 10.03 (0.62) | <0.001 | 10.05 (0.62) | <0.001 |
|  | Women (ref) |  |  | 0 |  | 0 |  |
|  | Cognitive status |  |  |  |  |  |  |
|  | Normal (≥26 MMSE score) |  |  | 1.85 (0.79 ) | 0.02 | 1.76 (0.80) | 0.03 |
|  | Impaired (ref) |  |  | 0 |  |  |  |
|  | Height |  |  | 0.18 (0.07) | 0.007 | 0.19 (0.07) | 0.007 |
|  | Fat-free mass |  |  | 0.14 (0.06) | 0.02 | 0.14 (0.06) | 0.01 |
|  | Physical activity |  |  |  |  |  |  |
|  | High |  |  | 3.37 (1.04) | 0.001 | 3.38 (1.04) | 0.001 |
|  | Moderate |  |  | 1.54 (0.98) | 0.11 | 1.51 (0.98) | 0.12 |
|  | Low (ref) |  |  | 0 |  | 0 |  |
|  | Decline |  |  |  |  |  |  |
|  | Time | -0.85 (0.08) | <0.001 | -1.3 (0.25) | <0.001 | -1.30 (0.25) | <0.001 |
|  | Slopes |  |  |  |  |  |  |
|  | Sex × Time |  |  |  |  |  |  |
|  | Men × Time |  |  | -0.62 (0.16) | <0.001 | -0.62 (0.16) | <0.001 |
|  | Women × Time (ref) |  |  | 0 |  | 0 |  |
|  | Physical activity × Time |  |  |  |  |  |  |
|  | High × Time |  |  | 0.83 (0.27) | 0.002 | 0.83 (0.27) | 0.002 |
|  | Moderate × Time |  |  | 0.77 (0.27) | 0.005 | 0.77 (0.27) | 0.005 |
|  | Low × Time |  |  | 0 |  | 0 |  |

*Parameter estimates β coefficients (SE) of fixed effects with GS longitudinal data. Random effects included both intercept and slopes of GS over 5 years. Time was used as continuous variable. Only significant fixed effects are reported.

^†^Low protein intake group was defined as having protein intake <1g/kg aBW/d. Women and low PA served as reference group.

Model 1 includes a linear trend of time.

Model 2 is additionally adjusted for sex, anthropometry (height and FFM), health-related factors (number of chronic diseases, self-rated health, cognitive impairment, arthritis in hands), PA, attrition variable, physical activity, and the interaction term (sex × time, PA × time).

Model 3 is further adjusted for protein distribution and food intake misreporting.

Supplementary Table 2. β coefficients* of growth curve models for Timed up-and-go (TUG) test over 5-year follow-up in low and good protein intake group^†^

| Outcome | Effects/variable | Model 1 |  | Model 2 |  | Model 3 |  |
| --- | --- | --- | --- | --- | --- | --- | --- |
|  |  | β (SE) | p | β (SE) | p | β (SE) | p |
| TUG (log_10_-s) | Intercept | 1.16 (0.01) | <0.001 | 1.52 (0.04) | <0.001 | 1.50 (0.05) | <0.001 |
| *Low protein* | Sex |  |  |  |  |  |  |
|  | Men |  |  | -0.05 (0.02) | 0.004 | -0.05 (0.02) | 0.005 |
|  | Women (ref) |  |  | 0 |  | 0 |  |
|  | Disease count |  |  | 0.02 (0.007) | 0.03 | 0.02 (0.007) | 0.03 |
|  | Self-rated health |  |  |  |  |  |  |
|  | Excellent/very good |  |  | -0.09 (0.02) | <0.001 | -0.09 (0.02) | <0.001 |
|  | Good |  |  | -0.09 (0.02) | <0.001 | -0.09 (0.02) | <0.001 |
|  | Fair/poor (ref) |  |  | 0 |  | 0 |  |
|  | Physical activity (PA) |  |  |  |  |  |  |
|  | High |  |  | -0.19 (0.03) | <0.001 | -0.19 (0.03) | <0.001 |
|  | Moderate |  |  | -0.11 (0.03) | <0.001 | -0.11 (0.03) | <0.001 |
|  | Low (ref) |  |  | 0 |  | 0 |  |
|  | Use of walking aids (No) |  |  | -0.15 (0.02) | <0.001 | -0.15 (0.02) | <0.001 |
|  | Yes (ref) |  |  | 0 |  | 0 |  |
|  | Decline |  |  |  |  |  |  |
|  | Time | 0.06 (0.006) | <0.001 | 0.06 (0.01) | <0.001 | 0.06 (0.01) | <0.001 |
|  | Time^2^ | -0.01 (0.001) | <0.001 | -0.01(0.001) | <0.001 | -0.01 (0.001) | <0.001 |
| TUG (log_10_-s) | Intercept | 1.11 (0.01) | <0.001 | 1.35 (0.05) | <0.001 | 1.33 (0.05) | <0.001 |
| *Good protein* | Disease count |  |  | 0.02 (0.007) | 0.001 | 0.02 (0.008) | 0.001 |
|  | Cognitive status |  |  |  |  |  |  |
|  | Normal (≥26 MMSE score) |  |  | -0.06 (0.02) | 0.02 | -0.06 (0.03) | 0.02 |
|  | Impaired (ref) |  |  | 0 |  |  |  |
|  | Physical activity |  |  |  |  |  |  |
|  | High |  |  | -0.14 (0.03) | <0.001 | -0.14 (0.03) | <0.001 |
|  | Moderate |  |  | -0.04 (0.03) | 0.18 | -0.04 (0.03) | 0.18 |
|  | Low (ref) |  |  | 0 |  | 0 |  |
|  | Use of walking aids (No) |  |  | -0.13 (0.02) | <0.001 | -0.13 (0.02) | <0.001 |
|  | Yes (ref) |  |  | 0 |  |  |  |
|  | Decline |  |  |  |  |  |  |
|  | Time | 0.06 (0.006) | <0.001 | 0.06 (0.01) | <0.001 | 0.06 (0.01) | <0.001 |
|  | Time^2^ | -0.008 (0.001) | <0.001 | -0.008 (0.001) | <0.001 | -0.008 (0.001) | <0.001 |

*Parameter estimates β coefficients (SE) of fixed effects with TUG longitudinal data (log_10_-transformed). Random effects included both intercept and slopes of TUG times (log_10_-transformed) over 5 years. Time was used as continuous variable. Only significant fixed effects are reported.

^†^Low protein intake was defined as having protein intake <1g/kg aBW/d.

Model 1 includes a linear and quadratic trend of time.

Model 2 is additionally adjusted for sex, anthropometry (height and FFM), health-related factors (number of chronic diseases, self-rated health, cognitive impairment), PA, attrition variable, use of walking aids at baseline and follow-up, and interaction term (PA × time in *Good protein* and sex × time in *Low protein* intake group).

Model 3 is further adjusted for protein distribution and food intake misreporting.
